# Anemia among Adolescent Girls Attending the Pediatric Outpatient Department of a Tertiary Care Hospital: A Descriptive Cross-sectional Study

**DOI:** 10.31729/jnma.6897

**Published:** 2021-09-30

**Authors:** Bikash Bhandari, Anuja Kachapati, Kavita Lamichhane, Gaurab Khadka

**Affiliations:** 1 Department of Pediatrics, Lumbini Medical College, Palpa, Nepal; 2 Department of Nursing, Universal College of Medical Sciences, Bhairahawa, Nepal; 3 Devdaha Medical College and Research Institute, Rupandehi, Nepal

**Keywords:** *adolescent*, *anemia*, *body mass index*

## Abstract

**Introduction:**

Adolescents are children aged 10-19 years. Nutrition influences the growth and development during infancy, childhood and adolescence. Adolescent girls are at higher risk of anemia and undernutrition. This research was aimed to find the prevalence of anemia among adolescent girls in a tertiary care hospital.

**Methods:**

A descriptive cross-sectional study was done in the adolescent girls attending the pediatric outpatient department of a tertiary care centre from October 2020 to May 2021. After the ethical clearance from the institutional review committee, 380 adolescent girls were taken using a convenient sampling technique. Anthropometric measurements, social demography and blood for hemoglobin estimation were taken and documented in predesigned proforma. Data were analyzed with Statistical Package for the Social Sciences version 20. Point estimate at 95% Confidence Interval was done, and frequency and proportion were calculated.

**Results:**

Out of 380 adolescent girls, 230 (60.5%) at 95% Confidence Interval (55.56-65.41) were anemic with mean hemoglobin of 11.138±1.954 gm/dl. The mean age was 14.57±2.107 years.

**Conclusions:**

This study showed a higher prevalence of anemia than the national data. Proper education regarding personal and menstrual hygiene, weekly supplementation of iron in school, dietary habits and uplifting of economic status can prevent anemia in this population.

## INTRODUCTION

Adolescence is derived from a Latin word 'adolescere' which means 'to grow up'.^1^ World Health Organization (WHO) defines adolescents as children aged 10-19 years of age.^2^ Around 6.38 million population (22% of total population) in Nepal are adolescent.^3^ Adolescent growth spurt results in 15% increase in iron requirements and girls being the most vulnerable.^4^

Anemia is a condition where the red blood cells and their oxygen carrying capacity is insufficient to meet the physiological needs of the body.^5^ The diagnosis of anemia is based on clinical features like fatigability, lethargy and pallor. During the adolescence period, requirement for iron doubles in girls as they lose iron during menstruation.^6^ Fewer studies have shown the higher prevalence of anemia in adolescent girls ranging from 42-60%.^7^

The objective of the study was to identify the prevalence of anemia in the adolescent girls in the tertiary care center of western Nepal.

## METHODS

This descriptive cross-sectional study was conducted in the pediatric outpatient department of Devdaha Medical College and Research Institute (DMCRI) from 15th October 2020 to 15th May 2021 after taking ethical clearance letter (034/2020) from the Institutional Review Committee of DMCRI. The present study included the adolescent girls attending the pediatric department of DMCRI between age group 10-19 years and those girls diagnosed as iron deficiency anemia under iron supplements; diagnosed with chronic hematological disorders like thalassemia, leukemia, sickle cell anemia, hemophilia and not willing to participate in the study were excluded. Data regarding anthropometric measurements (height and weight), hemoglobin levels and social demography were collected after taking informed consent and recorded in the predesigned proforma. Anemia was classified among the adolescent girls as per WHO classification.5 Convenience sampling was done and sample size was calculated using the following prevalence formula:

n = Z^2^ × p × q / e^2^

  = (1.96)^2^ × 0.5 × (1 - 0.5) / (0.06)^2^

  = 267

where,

n = sample size
Z = 1.96 at 95% Confidence Interval (CI)
p = prevalence of anemia in adolescent girls taken as 50% for maximum sample size
q = 1-p
e = margin of error 6%

Taking 10% non response rate, the sample size is 294. However, total sample size taken was 380. The data were recorded and analyzed using Statistical Package for the Social Sciences (SPSS) 20 software. Point estimate at 95% confidence interval (CI) was calculated along with frequency and proportion for binary data.

## RESULTS

**Figure 1:**
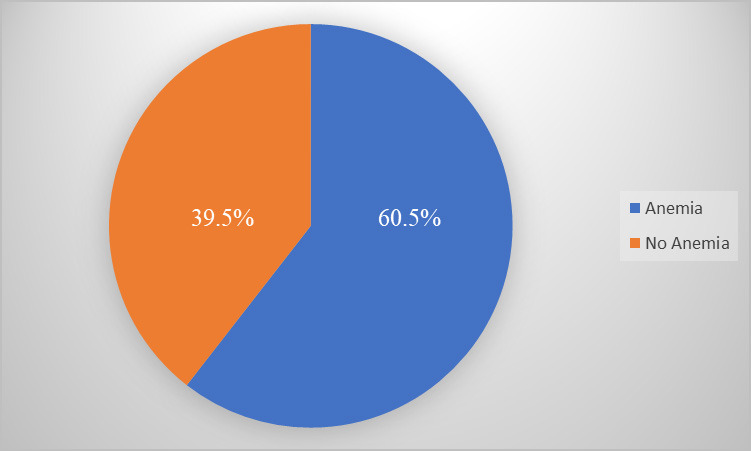
Prevalence of anemia in study population (n=380).

Among the 380 adolescent girls, 230 (60.5%) at 95% Confidence Interval (55.56-65.41) were anemic. Of these 55 (14.5%), 155 (40.7%) and 20 (5.3%) had mild, moderate and severe anemia respectively as per WHO classification. Similarly, 318 (83.7%) of the girls had normal Body Mass Index (BMI) with 49 (12.9%) and 13 (3.5%) above and below the normal range respectively (Figure 1, Table 1).

**Table 1:** Respondents' classification of anemia and body mass index (n=380).

Variables	n (%)
**Classification of anemia**	
Normal	150 (39.5)
Mild anemia	55 (14.5)
Moderate anemia	155 (40.7)
Severe anemia	20 (5.3)
**Classification of Body Mass Index (BMI)**	
Obesity	3 (0.8)
Overweight	46 (12.1)
Normal	318 (83.7)
Thinness	12 (3.2)
Severe thinness	1 (0.3)
Mean of weight= 48.76kg	

Two hundred and three (53.4%) were between 1014 years and 177 (46.6%) were between 15-19 years with mean age of 14.57±2.107 years. Among these, 318 (83.7%) had only basic education. Similarly, 294 (77.4%) had attained menarche and 318 (83.7%) were non-vegetarian by diet. Among the parents, 361 (95%) of the fathers and 227 (59.7%) of the mothers had formal education. About half of the mothers 193 (50.8%) were homemakers and 312 (82.1%) of the family had their monthly income between Rs.10,000 to Rs.50,000 (Table 2).

**Table 2:** Socio-demographic Profiles(n = 380).

Sociodemographic Variables	n (%)
**Age**	
10-14 years	203 (53.4)
15-19 years	177 (46.6)
Mean=14.57, SD=± 2.107	
**Standard of study**	
Basic education	318 (83.7)
Higher education	62 (16.3)
**Type of family**	
Nuclear	250 (65.8)
Joint	116 (30.5)
Extended	14 (3.7)
**Type of diet**	
Veg	62 (16.3)
Non-veg	318 (83.7)
**Number of Child**	
One	22 (5.8)
Two	173 (45.5)
Three	134 (35.3)
More than three	51 (13.4)
**Birth order**	
First child	185 (48.7)
Second child	140 (36.8)
Third child and more	55 (14.5)
**Attained menarche**	
Yes	294 (77.4)
No	86 (22.6)
**Educational status of mother**	
No formal education	153 (40.3)
Formal education	227 (59.7)
**Occupation of father**	
Government employee	44 (11.6)
Private employee	95 (25.0)
Self-employee	141 (37.1)
Daily wages	100 (26.3)
**Occupation of Mother**	
Homemaker	193 (50.8)
Government employee	24 (6.3)
Private employee	34 (8.9)
Self-employee	87 (22.9)
Daily wages	42 (11.1)
**Monthly family income**	
Rs. <10000	17 (4.5)
Rs. 10,000-25,000	152 (40.0)
Rs. 26,000-50,000	143 (37.6)
Rs. >50,000	68 (17.9)

The mean value of hemoglobin and BMI were 11.138gm/dl (10.913-11.346 at 95% CI) and 20.393kg/m^2^ (20.146-20.64 at 95% CI) respectively. Standard deviation of hemoglobin and BMI were 1.954 and 2.483 respectively.

Among the girls aged 10-14 years, 116 (57.1%) were anemic while 114 (64.4%) were anemic in the girls aged 15-19 years. Anemia was highly prevalent among the vegetarian girls 47 (75.8%) as compared to nonvegetarian girls 183 (57.5%). Similarly, prevalence of anemia was increased with higher birth order of the girls and higher number of siblings in the family. With increasing monthly income of the family, there was a decreasing trend in the prevalence of anemia in the adolescent girls (Table 3).

**Table 3:** Prevalence of anemia in demographic variables (n=380).

Sociodemographic variables	Anemia		Total (n = 380)
	Present (%)	Absent (%)	
**Age**			
10-14 years	116 (57.1)	87 (42.9)	203
15-19 years	114 (64.4)	63 (35.6)	177
**Type of family**			
Nuclear	154 (61.6)	96 (38.4)	250
Joint	71 (61.2)	45 (38.8)	116
Extended	5 (35.7)	9 (64.3)	14
**Type of diet**			
Veg	47 (75.8)	15 (24.2)	62
Non-veg	183 (57.5)	135 (42.5)	318
**Number of children**			
One	10 (45.5)	12 (54.5)	22
Two	108 (62.4)	65 (37.6)	173
Three	76 (56.7)	58 (43.3)	134
More than three	36 (70.6)	15 (29.4)	51
**Birth order**			
First child	108 (58.4)	77 (41.6)	185
Second child	84 (60.0)	56 (40.0)	140
Third child and more	38 (69.1)	17 (30.9)	55
**Attained menarche**			
Yes No	185 (62.9) 45 (52.3)	109 (37.1) 41 (47.7)	294 86
**Educational status of mother**			
Illiterate	106 (69.3)	47 (30.7)	153
Literate	124 (54.6)	103(45.4)	227
**Occupation of father**			
Government employee	30 (68.2)	14 (31.8)	44
Private employee	45 (47.4)	50 (52.6)	95
Self-employee	90 (63.8)	51 (36.2)	141
Daily wages	65 (65.0)	35 (35.0)	100
**Occupation of Mother**			
Homemaker	128 (66.3)	65 (33.7)	193
Government employee	15 (62.5)	9 (37.5)	24
Private employee	21 (61.8)	13 (38.2)	34
Self-employee	49 (56.3)	38 (43.7)	87
Daily wages	17 (40.5)	25 (59.5)	42
**Family income**			
Rs. <10000	12 (70.6)	5 (29.4)	17
Rs. 10,000-25,000	113 (74.3)	39 (25.7)	152
Rs. 26,000-50,000	79 (55.2)	64 (44.8)	143
Rs. >50,000	26 (38.2)	42 (61.8)	68

## DISCUSSION

The prevalence of anemia among the adolescent girls included in our study was 60.5%. This was higher than the national data of our country as per NDHS 2016 which showed it to be 44%.^8^ In contrast, findings from a nationally representative cross sectional survey done by Chalise B et al. showed the prevalence of anemia to be 38% among the adolescent females.^9^ The same study also showed higher prevalence in the population of terai as compared to hills and mountains which could be the reason of higher prevalence in our study.^9^ Studies done in the adolescent girls of Morang, Nepal and Maharashtra, India showed anemia in 51.3% and 60% of them respectively.^10-11^

Socioeconomic and demographic factors have a role in prevalence of anemia. Higher prevalence of anemia was seen among vegetarian girls in our study as compared to non-vegetarians. This was consistent with the study done by Dutt R, et al. where anemia was significantly higher with the vegetarians.^12^ This could be due to the higher bioavailability of heme iron from mixed diet despite its absorption being enhanced by vitamin C and inhibited by calcium and phytates.

There was a higher prevalence of anemia among the postmenarchal adolescents (62.9%) as compared to premenarchal girls (52.3%) in this study. This was in corroboration with the studies done in Karnataka and Maharashtra, India where the prevalence of anemia was higher among the postmenarchal girls, 71% and 90.65% respectively.^13-14^ This could be due to variable amount of blood flow during menstruation and lack of proper menstrual hygiene. However, this was discordant with the study done in Vellore, south India which showed no association between anemia and menarche.^15^

The socioeconomic factors and lack of education have been one of the major contributors for the presence of anemia in developing countries like Nepal.^9-11,16^ This too was consistent in our study where education of mother, occupation of both the parents and monthly income were the major determinants for the development of anemia in the studied adolescent girls. The reason could be the lack of awareness due to poor education, poor sanitation and hygiene and inadequate iron supplementation.

The major limitation of our study is that the adolescent boys are not included in the study. As the study population are taken from the Terai belt close to the Indian border, this does not represent the actual national data as the prevalence of anemia is higher in terai in comparison to hills and mountains.

## CONCLUSIONS

The prevalence of anemia was found to be higher in this study as compared to the national data. This is probably due to the small group of population taken from the Terai region where the prevalence of anemia is higher itself. Socioeconomic status, dietary habit, education status and occupation of the parents were one of the contributors for the development of anemia in the girls. Anemia not only affects the immediate health conditions but is also responsible for future reproductive morbidity and mortality. Weekly iron supplementation in school, regular training and monitoring regarding maintenance of personal and menstrual hygiene, and appropriate health education will help to lower the prevalence of anemia in our community.
